# A Comprehensive Evaluation of Thigh Mineral-Free Lean Mass Measures Using Dual Energy X-Ray Absorptiometry (DXA) in Young Children

**DOI:** 10.3390/jimaging11110374

**Published:** 2025-10-25

**Authors:** Trey R. Naylor, Mariana V. Jacobs, Michael A. Samaan, Laura C. Murphy, Douglas J. Schneider, Margaret O. Murphy, Hong Huang, John A. Bauer, Jody L. Clasey

**Affiliations:** 1 Department of Kinesiology and Health Promotion, University of Kentucky, Lexington, KY 40506, USA; trna226@uky.edu (T.R.N.); mariana.jacobs@uky.edu (M.V.J.); michael.samaan@uky.edu (M.A.S.); 2 Department of Pediatrics, University of Kentucky, Lexington, KY 40506, USA; laura.murphy2@uky.edu (L.C.M.); douglas.schneider@uky.edu (D.J.S.); maggie.murphy@uky.edu (M.O.M.); hong.huang@uky.edu (H.H.); john.bauer@uky.edu (J.A.B.)

**Keywords:** body composition, pediatrics, mineral-free lean, region of interest

## Abstract

This study aimed to (1) demonstrate the intra- and interrater reliability of quadriceps (QUADS) and hamstring (HAMS) mineral-free lean (MFL) mass measures using DXA scanning, (2) determine the association of total thigh MFL mass measures with MFL mass measures of the hamstrings and quadriceps combined and (3) analyze the association between total thigh MFL mass and total body MFL mass measures. A total of 80 young children (aged 5 to 11 yrs) participated and unique regions of interest were created using custom analysis software with manual tracing of the QUADS, HAMS, and total thigh MFL mass measures. Repeated-measure analysis of variance was used to determine if there were significant differences among the MFL measures while intraclass correlation coefficients (ICC), coefficients of variation (CV), and regression analysis were used to determine the intra- and interrater reliability and the explained variance in the association among MFL mass measures. The right interrater QUADS MFL mass was the only significant group mean difference, and ICCs between (≥0.961) and within (≥0.919) raters were high for all MFL measures with low variation across all MFL measures (≤6.13%). The explained variance was 92.5% and 96.3% for the between-investigator analyses of the right and left total thigh MFL mass measures, respectively. Furthermore, 97.5% of the variance in total body MFL mass was explained by the total thigh MFL mass. DXA MFL mass measures of the QUADS, HAMS and total thigh can be confidently used in young children and may provide an alternative to CT or MRI scanning when assessing changes in MFL cross-sectional area or volume measures due to disease progression, training and rehabilitative strategies.

## 1. Introduction

Originally introduced in 1987, dual-energy X-ray absorptiometry (DXA) has become a widely available and clinically useful tool in the evaluation and management of bone diseases [1]. While initially established to produce total body and regional bone mineral content (BMC; g) and bone mineral density content (BMD; g/cm^2^) measures [2,3], DXA has undergone recent advances in software and technological innovations that have allowed the inclusion of soft tissue analyses, including the absolute and relative fat and mineral-free lean (MFL) masses [4]. Furthermore, these advances in software developments, including the incorporation of a custom analysis function, contributes to the infinite possibilities for creating unique, custom regions of interest (ROI) for both bone and soft tissue analyses.

The use of DXA in the pediatric population has substantially increased in recent years [2,5]. DXA is preferred over other densitometric techniques for children because of its rapid measurement time (a total body scan requires approximately 10 min to perform), minimal radiation exposure to participants (less than 1 mrem for a total body scan), low cost, and widespread availability [6,7,8,9]. In addition, DXA and other methods of determining body composition may be more informative than using body mass index (BMI) percentiles or BMI z-scores to classify disease risk in children [10]. Total body DXA scans can generate automated and standardized ROI which separates the participants’ scans into axial and appendicular sections [11,12,13]. While useful diagnostic information is provided from these scans, specific body segment parameters and custom ROIs are not routinely provided and require additional operator analyses manipulation [14].

Total body DXA scans completed in the standard anterior–posterior positioning provide measures of the bone and soft tissue measures of the thigh using the custom analysis function. However, alternative positioning for total body scans is required if separate analyses of MFL masses of the lower extremities, specifically the quadriceps (QUADS) and hamstrings (HAMS), are desired. Furthermore, while using the total body scan mode, participants must lie on their right or left sides, with the measured leg extended and lying against the scan table in order to accomplish the alternative positioning for the QUADS and HAMS MFL mass measures. Since creating these unique ROIs requires subjectivity while tracing the QUADS and HAMS musculature, both intra- and interrater reliability measures should be determined [15,16,17]. While this unique thigh MFL mass and resulting reliability measures have been previously reported in adults [15,17], we are not aware of published reports including this type of analysis in pediatric populations. Furthermore, the association of MFL mass measures of the total thigh with the combined QUADS and HAMS MFL mass measures can also be determined and has not been reported in either adult or pediatric populations.

Reliable quantification of upper and lower extremity segmental MFL masses is very important when objectively examining changes in these masses over time [18,19] or when trying to determine the association to total body MFL mass. Prior studies in adults have demonstrated several methodological inconsistencies including variability in segmental boundaries, lack of uniformity in participant positioning during scanning, and discrepancies during post-scan analyses [11,19,20]. Additionally, total body MFL mass measures via DXA scanning have been used to quantitatively represent total skeletal muscle mass despite known assumption violations and an inability to distinguish among skeletal muscle mass, viscera and fluids [21].

Due to the lack of published reports concerning custom DXA MFL mass measures in pediatric populations, we sought to comprehensively evaluate DXA thigh MFL mass measures by (1) demonstrating the within (intra)-rater and between (inter)-rater reliability of QUADS and HAMS MFL mass measures using DXA, (2) determining the association of total thigh MFL mass measures with MFL mass measures of the QUADS and HAMS combined, and (3) analyzing the association between total thigh MFL mass measures and total body MFL mass measures.

## 2. Materials and Methods

**Subjects:** A convenience sample of 27 (13 males) young children (aged 5 to 11 yrs) completed the requirements for this study and were used for specific aims 1 and 2 to demonstrate the within (intra)-rater and between (inter)-rater reliability and to determine the association of total thigh MFL mass measures to MFL mass measures of the QUADS and HAMS combined. Data from a prior study was combined with the current convenience sample data (*n* = 80; 38 males), including 2 Black Non-Hispanic males, 2 Hispanic males, 2 Black Non-Hispanic females, 2 Biracial females, 2 Hispanic females and the remaining children identifying as White, Non-Hispanic, to assess specific aim 3, which was designed to analyze the association between total thigh MFL mass measures and total body MFL mass measures. Previously placed hardware interfering with body composition measures or a positive pregnancy test prior to DXA scanning were considered exclusion criteria for this study. Prior to participation in this study, parental written informed consent and verbal assent were obtained for each participant in accordance with the policies and procedures of the University of Kentucky Office of Research Integrity (Lexington, KY). The study was reviewed and approved by the University of Kentucky Medical Institutional Review Board (IRB) to ensure that the design of this study protected the rights of the participants.

**Anthropometric Measures:** Participants’ height and weight were determined using an electronic digital scale (BWB-800; Tanita Corporation, Tokyo, Japan) and a wall-mounted stadiometer (Healthometer Professional; Model 597KLPELSTAR; Alspin, IL, USA). Standing height was determined to the nearest 0.1 cm while body mass was determined to the nearest 0.01 kg. All participants were measured in light-weight clothing containing no metal and without shoes.

**Body Composition Measures:** Body composition measures were obtained using a GE Lunar iDXA bone densitometer (Lunar Inc., Madison, WI, USA) during a single testing session. Participants completed 3 DXA scans using the total body scan mode. In accordance with state and university policies and procedures, all female participants who achieved menarche were required to complete a urine pregnancy test (McKesson Corp., San Francisco, CA, USA) immediately prior to DXA scanning. All DXA scans were performed by a single trained investigator and analyzed using GE Lunar software version 14.10.

**Figure 1:**
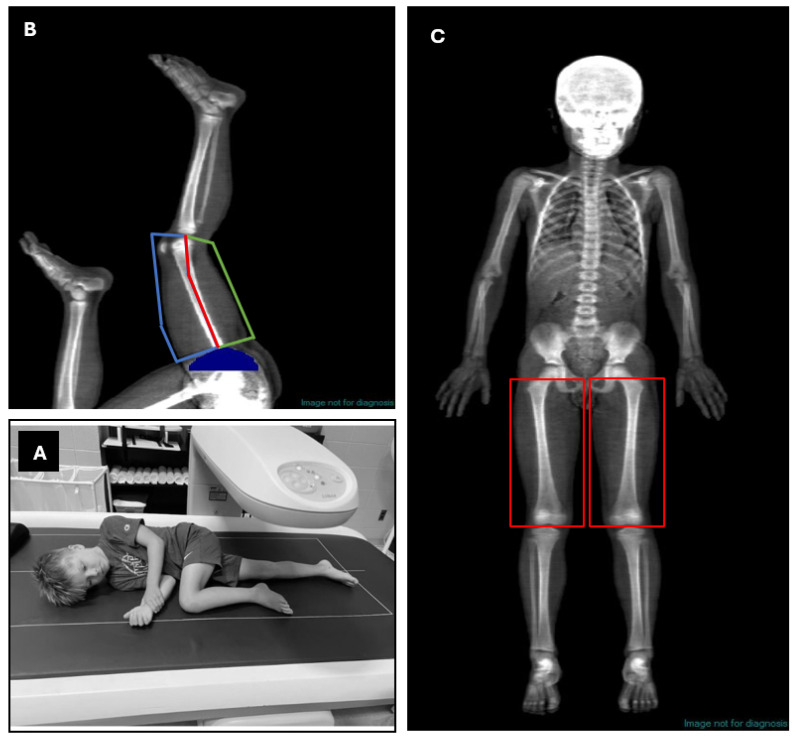
(**A**) Lateral subject positioning, (**B**) segmented body scan in the lateral view with ROI boxes, and (**C**) AP view using ROI boxes.

Participants performed the first DXA scan while lying in the standard anterior/posterior scanning position using the total body scan mode. This scan was then analyzed by the DXA scanning technologist to provide demographic information including total body fat-free and MFL masses and the absolute and relative fat masses. In addition, this total body DXA scan was used to determine the right and left total thigh MFL masses. Anatomical borders for these total thigh MFL masses were defined proximally as the top of the greater trochanter and distally intersecting the knee joint (Figure 1C). This scan was immediately followed by 2 additional scans using the total body scan mode while the participants were lying on their left and right sides with the leg of interest extended and the contralateral leg bent to avoid interference from the analysis field of view (Figure 1A). Furthermore, to reduce scan time and radiation exposure (albeit minimal) the final two DXA scans were performed with the participants lying upside down on the scan table. These scans started at the feet and were terminated when the scan reached the top of the iliac crests [17].

Custom ROI soft tissue analyses of MFL masses were created using analysis software that is available with iDXA (software version 14.01), with manual tracing of the QUADS and HAMS of the right and left thighs. The center of the femur (middle of the femoral shaft) and soft tissue borders were identified for the medial and lateral ROI while the base of the gluteal fold and knee joint were determined for the proximal and distal ROI borders, respectively (Figure 1B). To examine intra- and interrater reliability the MFL mass measures of the left and right QUADS and HAMS, determined using custom ROI, were analyzed twice by two investigators (Invest 1 and Invest 2) operating independently, without knowledge of each other’s results.

**Statistical Analysis:** Data were analyzed using statistical analysis software (SPSS, Version 23.0, Chicago, IL, USA) and significance was set at *p* < 0.05. For the original sample (*n* = 27), means, standard deviations (SD), and ranges for age, weight, height, body mass index (BMI), total body fat percentage (%fat), total body fat mass (FM), total body fat-free mass (FFM), total body MFL mass, and the fat-mass index (FMI) and fat-free-mass index (FFMI) [10,22] were determined using descriptive statistics. For the combined sample (*n* = 80), similar descriptives were used, but with the addition of total body MFL mass and total thigh MFL mass. Descriptive statistics were also used in the original sample to determine the mean, SD, and ranges for the MFL mass of the right and left QUADS and HAMS, as well as the right and left total thigh MFL mass for each investigator.

For the original sample (*n* = 27), a series of between-subject analyses of variance (ANOVA) were used to evaluate mean differences among the MFL measures of the right QUADS and HAMS, left QUADS and HAMS, right total thigh, and left total thigh within and between Invest 1 and Invest 2. Bland–Altman plots (Bland–Altman, 1986) were used to visually assess agreement while correlational analyses were used to determine the association by combining Invest 1 and Invest 2 right thigh mean versus right HAMS and QUADS combined mean and left thigh mean versus left HAMS and QUADS combined mean. Additionally, directional bias in Bland–Altman plotting was assessed by quantifying the association between method differences and averages using regression. Intra- and interrater reliability of the segmented scans was assessed using intraclass correlation coefficients (ICC) and coefficients of variation (CV). For the combined sample (*n* = 80), regression analyses were used to determine the association between total thigh MFL mass and total body MFL mass.

## 3. Results

A total of 80 participants (38 males) completed testing. Demographic, anthropometric, and body composition measures are presented in Table 1. Right and left QUADS and HAMS and right and left total thigh descriptives are displayed in Table 2. Within-group analyses for Invest 1 and Invest 2 revealed no significant mean differences for right and left QUADS and HAMS or right and left total thigh when comparing trial 1 to trial 2. Between-group analyses compared mean values for Invest 1 and Invest 2. Analyses revealed that Invest 1 had significantly higher means for right QUADS (775.8 ± 333.9 g vs. 723.1 ± 267.5 g) when compared to Invest 2.

The quantified measures of right and left QUADS and HAMS and right and left total thigh were used to assess reliability and are shown in Table 3. High interrater reliability was also demonstrated for right and left QUADS and HAMS and right and left total thigh. Interrater CV values demonstrated high reliability for right and left total thigh; however, slightly lower reliability and a larger CV value were demonstrated for right and left QUADS and HAMS. Segmented analyses resulted in strong intrarater reliability for right and left QUADS and HAMS and right and left total thigh (Table 3). Invest 1 demonstrated lower intrarater reliability than Invest 2 when comparing CV values; however, individual compartments including right and left QUADS and HAMS resulted in slightly lower reliability and larger CV values than right and left total thigh for Invest 1 and Invest 2.

**Figure 2:**
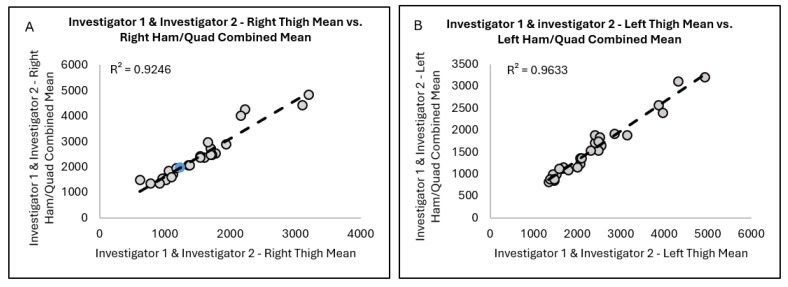
Associations between Investigator 1 and Investigator 2 combined (**A**) right thigh mean versus right HAM and QUAD combined mean and combined (**B**) left thigh mean versus left HAM and QUAD combined mean.

When examining the association between Invest 1 and Invest 2’s paired right thigh means and right QUADS and HAMS combined means (Figure 2A), we found that 92.5% of the variance in right thigh was explained by the right QUADS and HAMS combined. Similarly, when analyzing the association of Invest 1 and Invest 2’s paired left thigh means and left QUADS and HAMS combined means (Figure 2B), analysis showed that 96.3% of the variance in left thigh was explained by the left QUADS and HAMS combined.

**Figure 3:**
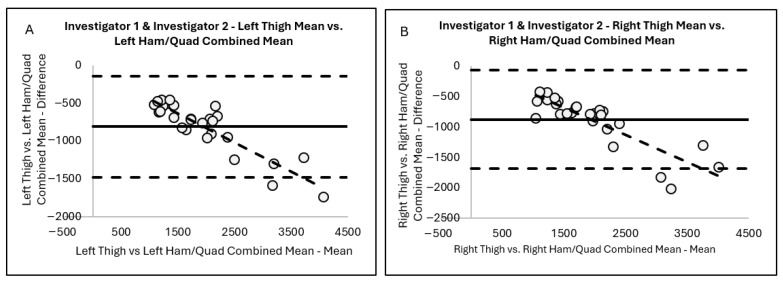
Bland–Altman plots for Investigator 1 and Investigator 2 combined (**A**) left thigh mean versus left HAM and QUAD combined mean and combined (**B**) right thigh mean versus right HAM and QUAD combined mean.

Bland–Altman plotting was used to visually assess the variability in the thigh MFL mass measurement analyses and the resulting mean difference, ±2 SD, for the right thigh versus the right QUADS and HAMS combined (−874.3 ± 412.4 g) (Figure 3A). In addition, Bland–Altman plotting demonstrated significant systematic (directional) bias suggesting that as the average of the two measures increases, the difference between the two measures trends in a negative direction for right thigh mean. The resulting mean difference, ±2 SD, for the left thigh versus the left QUADS and HAMS combined was −809.9 ± 340.9 g (Figure 3B). Similar to Figure 3A, the Bland–Altman plot indicated significant systematic (directional) bias suggesting that as the average of the measures increases, the difference between the two measures becomes progressively more negative for the left thigh mean.

When examining the association between total thigh MFL mass means and total body MFL mass means, 97.5% of the variance in total body MLF mass was explained by total thigh MFL mass.

## 4. Discussion

We have comprehensively evaluated the reliability of these unique regional MFL measures, and despite the smaller MFL masses examined, and the lack of employing a software ruler function or external marking devices [23], we showed excellent intra- and interrater reliability. To date, there have been very few studies that have determined the intra- and interrater reliability of QUADS and HAMS soft tissue measures when investigating custom ROI via DXA, and none of these studies included pediatric participants [15,16,17]. The current study is the first to report these reliability findings and the association between total thigh MFL mass and total body MFL mass in young children.

When comparing and contrasting reliability studies that similarly generated DXA thigh MFL measures, we found noteworthy differences among the study procedures and methodologies. Specifically, Raymond et al. [15] elevated the scanned leg at the ankle using a foam pad while an additional foam pad was used to ensure that the scanned leg of interest was straight. In addition, bone length measures were included to help reduce variability in measuring the custom created QUADS, HAMS and total thigh MFL mass measures. Additional procedural differences compared to our study included the soft tissue selected for inclusion within the ROI and determination of the proximal and distal borders (determined by leg length measures) [15]. When a DXA ruler function was not available as a software tool, Raymond-Pope and colleagues [23] used metallic markers placed on the skin as reference marks for creating the unique ROI [23]. Despite these methodological differences, the previous DXA-derived QUAD, HAMS and total thigh MFL mass reliability measures in adults [15,17] demonstrated similarly high intra- and interrater reliability measures when compared to the result of the current study conducted in young children. These finding were important to establish due to the smaller QUADS, HAMS and total thigh MFL mass measures in children, which were determined with identical pixel size or resolution used by the DXA analysis software.

The model and manufacturer used in this study was a GE Lunar iDXA (software version 14.10) which could be considered an additional reason for mixed outcomes. Furthermore, the impact of these factors may influence the ease and reliability of regional MFL measures. While differences in model and manufacturer may vary, a study conducted by Tombs and colleagues [24] reports that a correction within the GE Lunar iDXA magnification effect has improved resolution and image quality when determining unique ROIs [24].

A second aim of the current study was to determine the association of total thigh MFL mass measures to MFL mass measures of the QUADS and HAMS combined. While these analyses may seem unnecessary, due to the 2-D images created by the DXA scanner [25] and the subjectivity of the ROI created, we believe this is an important inclusion to comprehensively evaluate the thigh MFL measures. Based on the results of our nonsignificant group differences, and the high explained variance (92.5% and 96.3% for the right and left thigh, respectively), we can more confidently proceed with using these measures to demonstrate additional performance, clinical and rehabilitative associations. While our results suggest nonsignificant group differences and high explained variance, our Bland–Altman analysis indicated a systematic negative directional bias for both right and left thigh versus right and left QUADS and HAMS combined. We believe that the reason for this bias was in part the determination of the proximal border for the ROI lateral positioned scans and the proximal border of the right left HAMS MFL mass measures due to the lack of a bony landmark for delineation. These findings are similar to the findings of Naylor et al. [17] who also reported a high explained variance and a significant systematic negative directional bias for both right and left thigh versus right left QUADS and HAMS combined in young adults [17].

The final aim of this study was to explore the association between total thigh MFL mass measures and total body MFL mass measures. Correlational analysis and explained variance suggested that 97.5% of the variance in total body lean mass was explained by total thigh lean mass. Total and regional body composition assessment using MRI or CT scanning can provide both quantitative and qualitative assessment of tissue masses; however, they are cost-, time- and safety-prohibitive [26,27,28]. Alternatively, cross-sectional slices of the thigh using these MRI- or CT-based techniques have previously been used to represent baseline and changes in skeletal muscle cross-sectional area or volume due to training, treatment, and rehabilitation strategies [29,30,31,32]. More recently, the MFL mass measures via DXA scanning have been used to quantitatively represent total skeletal muscle mass despite known assumption violations and an inability to distinguish among skeletal muscle mass, viscera and fluids [33,34,35]. Our current findings suggest that thigh MFL mass is strongly associated with total body MFL mass in young children and may provide a useful regional DXA measure to evaluate the presence of characteristics of sarcopenia and the effectiveness of rehabilitative treatment strategies.

Clinically, a precise and accurate body composition measurement is desired when assessing disease progression and evaluating the effectiveness of intervention strategies [15,36]. Furthermore, body composition measurements via DXA scanning may be useful in children where other imaging techniques, including MRI and CT, remain a significant challenge in the pediatric population due to a longer scan time, claustrophobia, and increased exposure to radiation. The segmented lateral scanning method presented in this study could be used to assess and longitudinally monitor body composition changes in a variety of pediatric populations. In addition, monitoring changes in MFL mass measures over time may provide valuable insight concerning both normal and abnormal maturation and growth and help to evaluate young children for the presence of developmental sarcopenia [37]. Because the convenience sample was limited to children ages 5–11 years and we included children of varying adiposities and MFL masses, a limitation may include lack of generalizability to children outside of our age range or of specific adiposities. In addition, the results represent findings using GE Lunar iDXA software version 14.10 and may not be generalizable to other DXA manufacturers, models, and software versions.

In conclusion, obtaining QUADS and HAMS MFL measures using DXA may be a valuable tool to understand the association between strength, nutritional, and training status outcomes in young children. Furthermore, although MFL mass measures may be obtained using CT or MRI, DXA scan analysis has its advantages over these methods because of its rapid measurement time, minimal radiation exposure to participants, low cost, and widespread availability. Despite the same pixel size used for analyses by the DXA software analyses and the smaller thigh MFL mass measures of our pediatric population, our results demonstrated similar strong reliability when compared to studies performed in adults. Our results provide a very systematic and comprehensive evaluation of DXA thigh MFL mass measures in pediatric populations. Future studies should evaluate the stability and sensitivity of these DXA thigh MFL mass measures to track changes due to treatment, training, and rehabilitative strategies and their relationships to performance, nutrition and maturation. Specifically, our findings suggest that unique DXA scanning and analysis may be of great value when integrating screening to identify the presence or risks of developing sarcopenia. In addition, our findings may assist in providing information initiating early intervention and help determine the effectiveness of strategies to combat skeletal muscle loss [28,38]. The development of sarcopenia in pediatric populations may be associated with (but not limited to) cardiac abnormalities [39], congenital heart disease [40,41], transplantation [42], neuromuscular disease [43], and musculoskeletal morbidity due to interventional therapies [44]. Lastly, although our results revealed few group differences and strong reliability within and between investigators, we recommend that a single investigator should analyze the scans within a given study and use the mean of the two measures for final reporting.

## Figures and Tables

**Table 1:** Characteristics of study participants.

	Original Sample (*n* = 27)		Combined Sample (*n* = 80)	
	Mean ± SD	Range	Mean ± SD	Range
Age (yrs)	7.8 ± 2.0	5.1–11.9	8.9 ± 1.8	5.1–11.9
Weight (kg)	29.7 ± 10.9	18.3–58.4	34.1 ± 13.9	18.3–109.5
Height (cm)	129.0 ± 12.8	110.5–156.2	135.3 ± 13.1	110.5–173.9
Body Mass Index (kg.m)	17.3 ± 3.2	13.7–26.5	18.1 ± 4.2	10.3–36.2
Body Mass Index Percentile	57.9 ± 28.3	2.0–99.0	58.9 ± 30.4	2.0–99.0
Body Fat %	26.4 ± 6.1	18.1–42.6	27.4 ± 7.5	14.3–50.4
Fat Mass (kg)	8.2 ± 4.9	3.6–24.7	10.0 ± 7.3	3.2–51.4
Fat-Free Mass (kg)	20.4 ± 6.5	12.7–37.8	23.6 ± 7.2	12.7–53.9
Mineral-Free Lean Mass (kg)	19.4 ± 6.3	11.7–36.0	22.4 ± 6.9	11.7–51.1
Fat-Mass Index (kg/m^2^)	4.7 ± 1.9	2.6–10.3	5.2 ± 2.8	2.2–17.0
Fat-Free-Mass Index (kg/m^2^)	12.0 ± 1.7	9.8–17.6	12.6 ± 1.7	9.9–17.8

**Table 2:** QUADS, HAMS, and total thigh descriptives and ANOVA results (original sample; *n* = 27).

			Investigator 1						Investigator 2			
	Trial 1		Trial 2		Mean		Trial 1		Trial 2		Mean	
Compartment	Mean ± SD	Range	Mean ± SD	Range	Mean ± SD	Range	Mean ± SD	Range	Mean ± SD	Range	Mean ± SD	Range
Right Quadricep (g)	782.5 ± 344.4	363.0–1892.0	769.1 ± 324.7	373.0–1736.0	775.8 ± 333.9 ^a^	368.0–1814.0	723.2 ± 268.3	407.0–1434.0	723.0 ± 266.7	403.0–1425.0	723.1 ± 267.5	405.0–1429.5
Left Quadricep (g)	772.6 ± 286.3	409.0–1397.0	773.9 ± 293.5	408.0–1243.0	773.2 ± 289.5	408.5–1410.5	774.4 ± 343.2	368.0–1740.0	774.9 ± 340.3	372.0–1734.0	774.6 ± 341.7	370.0–1737.0
Right Hamstring (g)	773.7 ± 333.1	336.0–1708.0	771.1 ± 326.7	351.0–1720.0	772.4 ± 329.7	343.5–1714.0	767.0 ± 316.1	358.0–1609.0	766.3 ± 311.7	365.0–1608.0	766.7 ± 313.9	361.5–1608.5
Left Hamstring (g)	786.2 ± 350.0	382.0–1738.0	786.9 ± 346.3	379.0–1719.0	786.6 ± 347.9	380.5–1728.5	775.7 ± 323.1	375.0–1664.0	774.3 ± 320.6	391.0–1647.0	775.0 ± 321.8	383.0–1655.5
Right Total Thigh (g)	2380.1 ± 950.3	1340.0–4886.0	2371.1 ± 950.9	1321.0–4842.0	2375.6 ± 950.4	1331.5–4864.0	2372.7 ± 964.4	1314.0–4816.0	2371.1 ± 968.3	1316.0–4823.0	2371.9 ± 966.3	1315.0–4819.5
Left Total Thigh (g)	2378.6 ± 930.8	1341.0–4948.0	2367.8 ± 933.0	1356.0–4954.0	2373.2 ± 931.8	1348.5–4951.0	2356.4 ± 936.2	1342.0–4903.0	2359.6 ± 943.2	1357.0–4967.0	2359.0 ± 939.7	1349.5–4935.0

^a^ *p* < 0.05; between groups; Investigator 1 mean versus Investigator 2 mean. ‘Right Total Thigh’ and ‘Left Total Thigh’ refer to the sum of the QUADS and HAMS compartment for each measure of each leg (right and left). Abbreviations: SD = standard deviation.

**Table 3:** Interrater and intrarater reliability CV for quadriceps, hamstrings, and total thigh (original sample; *n* = 27).

	Intrarater Coefficients Interrater Coefficients					
	Investigator 1				Investigator 2	
Compartment	CV	ICC	CV	ICC	CV	ICC
Right Quadricep (g)	2.33	0.995	0.31	1.000	6.13	0.961
Left Quadricep (g)	1.18	0.997	0.33	1.000	5.45	0.969
Right Hamstring (g)	0.89	0.919	0.51	1.000	5.71	0.973
Left Hamstring (g)	1.14	0.999	0.35	1.000	3.54	0.989
Right Total Thigh (g)	0.53	1.000	0.23	1.000	1.08	0.999
Left Total Thigh (g)	0.39	1.000	0.27	1.000	0.64	0.999

‘Right Total Thigh’ and ‘Left Total Thigh’ refer to the sum of the quadricep and hamstring compartment for each measure of each leg (right and left). Abbreviations: CV = coefficient of variation; ICC = intraclass correlation coefficients.

## Data Availability

The original contributions presented in this study are included in the article. Further inquiries can be directed to the corresponding author.
